# Foodborne botulism due to ingestion of home-canned green beans: two case reports

**DOI:** 10.1186/s13256-017-1523-9

**Published:** 2018-01-04

**Authors:** Dorothea Hellmich, Katja E. Wartenberg, Stephan Zierz, Tobias J. Mueller

**Affiliations:** Neurointensive Care Unit, Department of Neurology, University Hospital Halle (Saale), Ernst-Grube-Strasse 40, D-06097 Halle (Saale), Germany

**Keywords:** Foodborne botulism, Botulism antitoxin, Respiratory failure, Autonomic disorders

## Abstract

**Background:**

Foodborne botulism is a life-threatening, rapidly progressive disease. It has an incidence of less than 10 cases per year in Germany and mostly affects several previously healthy people at the same time. The only specific treatment is the administration of botulism antitoxin. According to the German guidelines administration of antitoxin is recommended only in the first 24 hours after oral ingestion of the toxin.

**Case presentation:**

A 47-year-old white woman and her 51-year-old white husband presented with paralysis of multiple cranial nerves and rapidly descending paralysis approximately 72 hours after ingestion of home-canned beans. The disease was complicated by autonomic changes like hypertension, febrile temperatures, and a paralytic ileus. The diagnosis was confirmed by identification of botulinum neurotoxin type A in the serum of the woman. In accordance with the German guidelines, antitoxin was not given due to the prolonged time interval at diagnosis. Both patients had a long intensive care unit course requiring ventilation for approximately 5 months. Finally they recovered completely.

**Conclusions:**

A full recovery from foodborne botulism is possible even in patients with intensive care lasting several months. There are only case reports indicating that administration of antitoxin may shorten the course of the disease, even if given later than 24 hours after intoxication. Due to the rarity of the disease and its rapid course there are no randomized controlled trials. Thus, evidence of the superiority of this treatment is lacking. However, the prevailing view according to the German guidelines to administer antitoxin only within 24 hours after ingestion of the toxin should be questioned in the case of progression of the disease with proof of remaining toxin in the blood.

## Background

The incidence of botulism is less than 10 cases per year in Germany [1] and approximately 113 cases in the EU per year from 2008 to 2012 [2]. Of all forms of botulism including infant, wound, and adult intestinal toxemia botulism, foodborne botulism is the most frequent in Germany [1]. The diagnosis of foodborne botulism is made on high suspicion based on clinical findings of cranial nerve palsies and descending paralysis and a history of ingestion of food with low acid content in an anaerobic milieu, most commonly home-canned vegetables or meat. The diagnosis is confirmed by identification of botulinum neurotoxin (BoNT) in patients’ stomach or intestinal contents, vomit or feces, in blood in the hyperacute stage, or in the ingested food [3]. The ingested toxin can be demonstrated in serum or feces in 40 to 44% of cases within 3 days of toxin ingestion, and in 15 to 23% of cases in specimens obtained after 3 days [4].

After ingestion, BoNT, a preformed neurotoxin by *Clostridium botulinum*, binds to receptors in the presynaptic cell membrane. It crosses the membrane through endocytosis and acts as protease to the synaptic fusion complex. Therefore, the release of vesicles containing acetylcholine in the synaptic cleft is inhibited [4], followed by failure of neuromuscular transmission causing paralysis and autonomic disturbances. Improvement of muscle strength is caused by two factors. First, there is a sprouting of axonal collaterals from the presynaptic nerve endings at the neuromuscular junctions, which have the ability to form functional synapses. Second, later, synaptic activity returns to the original nerve terminals, and the sprouts are eliminated [5].

Therapy consists of supportive care and, if required, intensive care including long-term ventilation. A full recovery is possible, but the disease can have a long and exhausting course, and patients may experience and die from complications of intensive care medicine. The only specific therapy is the administration of botulism antitoxin. Trivalent (Anti-BoNT A, B, and E), equine-derived antitoxin, is available and neutralizes BoNT [3]. According to the guidelines in Germany, antitoxin should be given only within the first 24 hours after ingestion of the toxin [6]. This recommendation is based on a report of patients with wound botulism [7]. However, a retrospective analysis of 132 patients treated between 1973 and 1980 revealed that patients who received antitoxin later than 24 hours after onset of symptoms spent fewer days in hospital, fewer days on a ventilator, and fewer days to sustained improvement, than those who were not treated with antitoxin [8].

## Case presentation

### Case 1

A 47-year-old white woman presented to an outside hospital with subacute and progressive dizziness, diplopia, dysarthria, and bilateral ptosis, which started 8 hours prior to admission. Cranial computed tomography (CT) and magnetic resonance imaging (MRI) revealed normal results. A cerebrospinal fluid (CSF) analysis was normal. Her neurological symptoms worsened rapidly including a complete bilateral ptosis with inability to open her eyes, descending quadriparesis, and paralysis of her respiratory musculature. Approximately 24 hours following admission she required intubation and mechanical ventilation. Her husband presented with similar signs and symptoms a day later. Botulism was suspected, especially when the son remembered that his parents had eaten home-canned beans of unknown age 2 days prior to the admission of his mother. The son was present at dinner but refused to eat the beans because of an odd odor.

A mouse bioassay test with her serum revealed the presence of botulinum toxin A. Botulism antitoxin was not administered because more than 72 hours had passed since the ingestion of the probably poisoned beans. A tracheotomy was performed on hospital day 7; a percutaneous endoscopic gastrostomy (PEG) tube was placed on day 8. In addition, she developed gastrointestinal tract paralysis as well as anxiety. Both improved with symptomatic treatment. On hospital day 27 she was transferred to an acute rehabilitation hospital with complete paralysis of all cranial nerves and persisting quadriparesis: strength 2 to 3/5 Medical Research Council (MRC). She was alert and able to respond to questions with hand waving and writing. Finally, she was weaned from mechanical ventilation after 5.5 months and discharged from the rehabilitation hospital after 11 months. The neurological signs caused by botulism had completely resolved. However, she still felt weak and remained with a depressive adaptive disorder.

### Case 2

A 51-year-old white man, the husband of case 1, presented to an outside hospital with nausea, dizziness, progressive dysarthria, and diplopia which had started the day prior to admission. After transfer to our neurointensive care unit he showed bilateral ptosis, ophthalmoparesis with diplopia in all directions, dysarthria, dysphagia with disturbance of the oral and pharyngeal phase, and moderate bilateral facial nerve paralysis. He had full strength in his extremities and was able to walk normally. He had consumed a smaller portion of the home-canned green beans 3 days prior to admission.

Botulism antitoxin was not administered because of the prolonged time interval from ingestion of the presumed poisoned beans of more than 72 hours (Table 1). The remainder of the home-canned beans was found in the couple’s home and contained BoNT A. A mouse bioassay test with his serum was equivocal because only 50% of the mice developed symptoms of botulism. Neurological decline with progressing dysarthria, dysphagia, mydriasis, ophthalmoparesis, facial nerve paralysis, tongue paralysis, and development of a quadriparesis was observed within the next 3 days. A trial of neostigmine on hospital day 3 did not result in improvement. He was intubated and placed on mechanical ventilation on the same day for respiratory distress due to vomiting and an inability to clear secretions. Electrophysiological studies (including blink reflex and repetitive nerve stimulation, 3 Hz, of the facial nerve) on hospital day 5 were normal. The hospital course was complicated by aspiration pneumonia, delirium with a cycle of agitation and impaired level of consciousness, and uncontrollable hypertension unresponsive to intravenously administered antihypertensives and sedatives as well as central fever (no evidence of infection was found). He underwent tracheotomy on hospital day 7. He was transferred to an acute rehabilitation hospital on day 15 with mild quadriparesis (strength 4/5 MRC) and unchanged bilateral ptosis, mydriasis, ophthalmoparesis, bilateral facial paralysis, and tongue paralysis. While in acute rehabilitation, his quadriparesis worsened (strength 3/5 MRC) and a PEG was placed. Subsequently, recovery of strength started cranially and spread caudally. He was weaned from ventilation 4.5 months after ingestion of the toxin and was discharged home after 8 months without any neurological deficit, but with complaints of generalized weakness and muscle pain. At that time he reported complete amnesia and paranoid thoughts for the first 3 months of his hospital stay.

**Table 1:** Selected periods of Cases 1 and 2 in the disease course

	Ingestion of toxin to symptom onset, hours	Symptom onset to resp. failure, hours	Ingestion of toxin to resp. failure, hours	Duration of intubation, days
Patient 1 (female, age 47 years)	30	36	66	~165
Patient 2 (male, age 51 years)	48	82	130	~135

*resp.* respiratory

## Discussion

In both cases, diagnosis of foodborne botulism was suspected based on the typical signs, such as cranial nerve paralysis including diplopia, blurred vision, ptosis, dysarthria, and dysphagia, followed by descending, symmetrical muscle paralysis, affecting the respiratory muscles at an early stage [9] and on the information of ingestion of home-canned beans before admission.

The diagnosis was eventually confirmed by identification of BoNT type A in serum of case 1. Because of the early symptoms of diplopia and ptosis, other differential diagnoses of impaired neuromuscular transmission included myasthenia gravis and Lambert–Eaton syndrome. Nerve conduction studies and electromyography cannot significantly contribute to these differential diagnoses. A decremental response upon low frequency repetitive nerve stimulation might identify myasthenia gravis. However, in the early stages of the disease of botulism the response upon low frequency stimulation might be normal or only slightly decremental. High frequency repetitive nerve stimulation might show an increment, although smaller than in Lambert–Eaton syndrome. Thus, the normal electrophysiological findings in case 2 were not surprising, although high frequency stimulation was not performed [10, 11].

Case 1 and case 2 had a long intensive care unit course requiring ventilation for approximately 5 months and hospitalization for 11 and 8 months, respectively.

In addition, case 2 developed severe hypertension which was difficult to control as well as prolonged elevated temperature without signs of an infection. Both autonomic symptoms can be explained by an inhibition of the cholinergic parasympathetic nervous system. Autonomic symptoms due to botulinum toxin poisoning, including high resting heart rate, supine hypertension, orthostatic hypotension, and impaired baroreflex function were reported even in mild clinical courses [12]. Moreover, autonomic dysfunction can be the leading symptom of botulism type B [13].

As proscribed by the German guidelines [6], botulism antitoxin had not been given to our patients as the time interval from ingestion was approximately 72 hours at the time of diagnosis. Other recommendations, for example the botulism fact sheet of the World Health Organization (WHO), include that antitoxin should be administered as soon as possible, but they do not include restrictions due to the time interval between ingestion of the toxin and start of antitoxin administration (http://www.who.int/mediacentre/factsheets/fs270/en/).

Evidence for or against treatment with botulism antitoxin is low. There are no randomized controlled trials due to the rarity and character of the disease [14]. A previous study showed that specific therapy with botulism antitoxin may lead to shorter hospital stay and decreased time of mechanical ventilation also in patients who were treated more than 24 hours after ingestion of the toxin [15] (Table 2). Furthermore, in ten patients, antitoxin administration on day 4 after symptom onset resulted in reduced duration of mechanical ventilation compared to eight patients who received antitoxin on day 6, indicating a therapeutic benefit [15].

**Table 2:** Examples of recent incidences of foodborne botulism with special attention to time to application of antitoxin and duration of ventilation

Source of outbreak (state and year, type of botulinum toxin) Reference	Number of cases/Respiratory failure	Symptom onset to resp. failure, hours	Ingestion of toxin to resp. failure, hours	Ingestion of toxin to application of antitoxin, hours	Duration of intubation, days
Smoked ribs (China 2013, A) [16]	12/2	Patient 1: 26 Patient 2: 78	Patient 1: 58 Patient 2: 110	54	Patient 1: 12 Patient 2: 9
Bamboo shoots (Thailand 2006, A) [15]	209/42 (18 of 42 were followed up)	4–95	46–97	10 patients: 96 8 patients: 144	Antitoxin given after 96 hours: 10–23 days. Antitoxin given after 144 hours: 10–36 days
Carrot juice (USA 2006, A) [17]	6/6 (5 of 6 were treated with antitoxin)	8–96	38–72 (^a^)	Patient 1–3: 37–41 Patient 4: ~293 Patient 5: ~1057	Antitoxin given: 54 to > 365 (one patient died). Antitoxin not given: 129
“Pruno” home-canned wine (USA 2011, A) [18]	8/3	39–89	39–89	72–99	Patient 1: 6 Patient 2: 19 Patient 3: 80

^a^Two patients with unknown time of ingestion. *resp.* respiratory

In addition, there is a case report of less affected patients with no need for mechanical ventilation treated with botulism antitoxin up to 8 days after symptom onset, who fully recovered within 6 months [16]. It remains open if the treatment was still successful or the good clinical course was mainly driven by the probably low dosage of the toxin.

The botulism toxin was detectable in the blood of case 1 even more than 72 hours after toxin ingestion. The late detectability of the toxin in this patient is a strong argument to start an antitoxin therapy even in patients with ingestion of the toxin more than 72 hours ago. Case 2 showed a neurological deterioration within the first 6 days after ingestion of the toxin. An initial increase of the effect of the neurotoxin over days is seen in foodborne botulism, as well as after therapeutic toxin injections of BoNT. The reason for the prolonged deterioration is not clear. It might be a continuing mechanism on the synapses of BoNT or an effect of the sustained circulating toxin.

Altogether the latest time of effective antitoxin administration is unknown, but there are indications that it has a beneficial effect when given more than 24 hours after ingestion of toxin. Therefore the recommendation to administrate antitoxin should not be restricted to the first 24 hours after ingestion of toxin. Antitoxin should be administered to all patients within 72 hours after toxin ingestion; it should also be considered in cases of progression of the disease.

## Conclusions

This case report indicates that full recovery from foodborne botulism is possible even in patients with long-lasting intensive care for several months. The treatment with antitoxin may lead to shorter hospital stay and decreased time of mechanical ventilation and should therefore be considered at every stage of disease. It should not be restricted to the first 24 hours after the ingestion of toxin, as recommended in the German guidelines.

## Abbreviations

BoNT: Botulinum neurotoxin
PEG: Percutaneous endoscopic gastrostomy
MRC: Medical Research Council
